# Glymphatic System Dysfunction and its Associated Factors in Wilson's Disease: A DTI–ALPS Study

**DOI:** 10.1002/brb3.71658

**Published:** 2026-09-30

**Authors:** Ling Zhu, Tong Wu, Liang‐Jie Zhang, Qin‐yuan Liu, Liang‐liang Zhang, Bin Song, Yu‐long Zhu, Lei Hua, Long Zhang, Bo Li, Quan Sun, Ben‐chun Xue, Yin Xu, Yong‐zhu Han, Yong‐sheng Han

**Affiliations:** ^1^ Institute of Neurology Anhui University of Chinese Medicine Hefei China; ^2^ Affiliated Hospital of Institute of Neurology Anhui University of Chinese Medicine Hefei China; ^3^ Department of Radiology The Fourth Clinical Medical College of Guangzhou University of Chinese Medicine (Shenzhen Traditional Chinese Medicine Hospital) Shenzhen China; ^4^ Nanjing University of Chinese Medicine, Nanjing, China; ^5^ Wannan Medical College Wuhu China; ^6^ Center For Xin'an Medicine and Modernization of Traditional Chinese, Medicine of IHM Anhui University of Chinese Medicine Hefei China

**Keywords:** analysis along the perivascular space index, diffusion tensor imaging, glymphatic system, Wilson's disease

## Abstract

**Background:**

Wilson's disease (WD) is a genetic disorder of copper (Cu) metabolism that causes Cu accumulation in multiple organs, particularly the liver and brain, resulting in progressive multisystem damage. This study investigated glymphatic system function and its associated factors in patients with WD.

**Methods:**

Seventy‐one patients with WD (19 hepatic, 52 neurological) and 15 healthy controls (HCs) were enrolled. The analysis along the perivascular space (ALPS) index was quantified using diffusion tensor imaging (DTI–ALPS). Comparisons were performed between patients and than HCs, and across WD phenotypes, and correlations with clinical characteristics were analyzed.

**Results:**

Compared with HCs, patients with WD exhibited significantly reduced ALPS index (*p* < 0.001). Patients with neurological WD had a significantly lower ALPS index than those with hepatic WD (*p* = 0.018) and HCs (*p* < 0.001). No significant difference was observed between patients with hepatic WD and HCs (*p* = 0.077). In neurological WD, the ALPS index was significantly higher on the left side than that on the right (*p* < 0.001), whereas no significant interhemispheric differences were found in hepatic WD or HCs (*p* > 0.05). In neurological WD, the ALPS index correlated positively with albumin, fasting blood glucose, and the white matter volume‐to‐total intracranial volume ratio (V_WM/TIV ratio), and negatively with age, alanine aminotransferase, aspartate aminotransferase (AST), and the unified WD rating scale‐part I (UWDRS‐I) score. Multiple regression identified age, AST, and the UWDRS‐I score as independent predictors of reduced ALPS index.

**Conclusions:**

Neurological WD was associated with impaired glymphatic system function and altered lateralization. Age, AST, and the UWDRS‐I score were independently associated with dysfunction.Glymphatic function, assessed by DTI–ALPS index, was impaired in neurological WD compared with the hepatic phenotype and controls. Age, AST, and UWDRS‐I score independently predicted dysfunction, linking neurodegeneration, liver injury, and altered glymphatic activity.

## Graphical abstract

Glymphatic function, assessed by DTI‐ALPS index, was impaired in neurological Wilson's disease compared with hepatic phenotype and controls. Age, AST, and UWDRS‐I score independently predicted dysfunction, linking neurodegeneration, liver injury, and altered glymphatic activity.

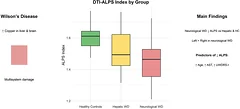

## 1 Introduction

Wilson's disease (WD) is an autosomal recessive copper (Cu) metabolism disorder caused by mutations in ATP7B. This genetic defect leads to excessive Cu accumulation in various organs and tissues, including the liver, brain, and kidneys, resulting in multisystem damage. The prevalence of WD ranges from 2.00–3.33 per 100,000 individuals in the United States, Europe, and Asia (Sandahl et al. 2020), whereas in some regions of mainland China, the reported prevalence is higher, between 4.93 and 6.21 per 100,000 (Hu et al. 2011; Hu et al. 2012). WD presents with highly heterogeneous clinical features, primarily affecting the liver and brain. The average age at onset of hepatic symptoms is between 10 and 13 years (Merle et al. 2007), whereas neurological symptoms typically manifest between 20 and 30 years (Dalvi 2014). Neurological involvement represents one of the most severe manifestations of WD and commonly includes movement disorders, psychiatric and behavioral abnormalities, and cognitive impairment (Wang et al. 2023).

The glymphatic system was first described by Iliff et al. in 2012 (Iliff et al. 2012). It is primarily composed of the perivascular space Virchow–Robin space (VRS), an aquaporin‐4 (AQP4) channel‐mediated cerebrospinal fluid‐interstitial fluid transport pathway at astrocytic endfeet, and a paravenous interstitial fluid drainage pathway. Its principal physiological function is the clearance of metabolic waste from the brain (Iliff et al. 2012). The role of the glymphatic system in neurological diseases has attracted increasing attention. Impaired glymphatic function has been reported in various neurodegenerative disorders, including Alzheimer's disease (Zhang et al. 2023; Huang et al. 2024), frontotemporal dementia (Xiao et al. 2024), Parkinson's disease (Huang et al. 2025; Miao et al. 2025), Huntington's disease (Yin et al. 2025), and amyotrophic lateral sclerosis (Huang et al. 2025, Baek et al. 2025), and is hypothesized to result from inadequate clearance of neuropathological proteins. Currently, glymphatic function is primarily assessed using magnetic resonance imaging (MRI) techniques, which are broadly categorized into those requiring contrast agents or tracers and those that are non‐invasive. The diffusion tensor imaging along the perivascular space (DTI–ALPS) technique is a non‐invasive method that has demonstrated good robustness (Taoka et al. 2022; Liu et al. 2024) and broad clinical applicability. It has been employed to evaluate glymphatic function in several neurological conditions, including Alzheimer's disease (Guo et al. 2025), idiopathic normal pressure hydrocephalus (Yang et al. 2025), Parkinson's disease (Huang et al. 2025), hyperglycemia (Zhang et al. 2025), and cerebral small‐vessel disease (Lu et al. 2025).

Based on this theoretical background, we hypothesized that excessive Cu accumulation may impair glymphatic system function in WD. Such impairment could impede Cu clearance and ultimately influence disease progression and prognosis. Therefore, this study employed the DTI–ALPS technique to characterize glymphatic system function in patients with WD and to identify potential influencing factors. We anticipate that this study will provide novel insights into the pathogenesis of WD and establish a theoretical basis for future therapeutic strategies.

## 2 Materials and Methods

### 2.1 Study Participants

Seventy‐one patients with WD were recruited from the inpatient department of a neurological specialty hospital in Anhui Province. The inclusion criteria were as follows: (1) meeting the diagnostic standards outlined in the 2022 American Association for the Study of Liver Diseases (AASLD) practice guidelines for WD, with a Leipzig score of ≥4 (Schilsky et al. 2023); (2) the ability to cooperate with and complete relevant brain MRI sequences; and (3) the provision of informed consent by the patient or their legal guardian. The exclusion criteria were: (1) contraindications to MRI scanning (e.g., a cardiac pacemaker, severe claustrophobia); (2) severe motor disorders or psychobehavioral abnormalities such as agitation, considered likely to prevent cooperation and stillness during scanning; (3) comorbidity with other diseases that could affect brain structure or function, such as encephalitis, traumatic brain injury, epilepsy, cerebrovascular diseases, diabetes, and hypertension; (4) incomplete clinical data; and (5) refusal or inability to provide informed consent. Additionally, 15 healthy controls (HCs) matched for age, sex, and education level were enrolled. All participants (patients and HCs) provided written informed consent before enrolment. The study protocol was approved by the Ethics Committee of my hospital (2023‐SYSFYSY‐32), and conducted in accordance with the Declaration of Helsinki. The participant screening flowchart is shown in Figure 1.

**FIGURE 1:**
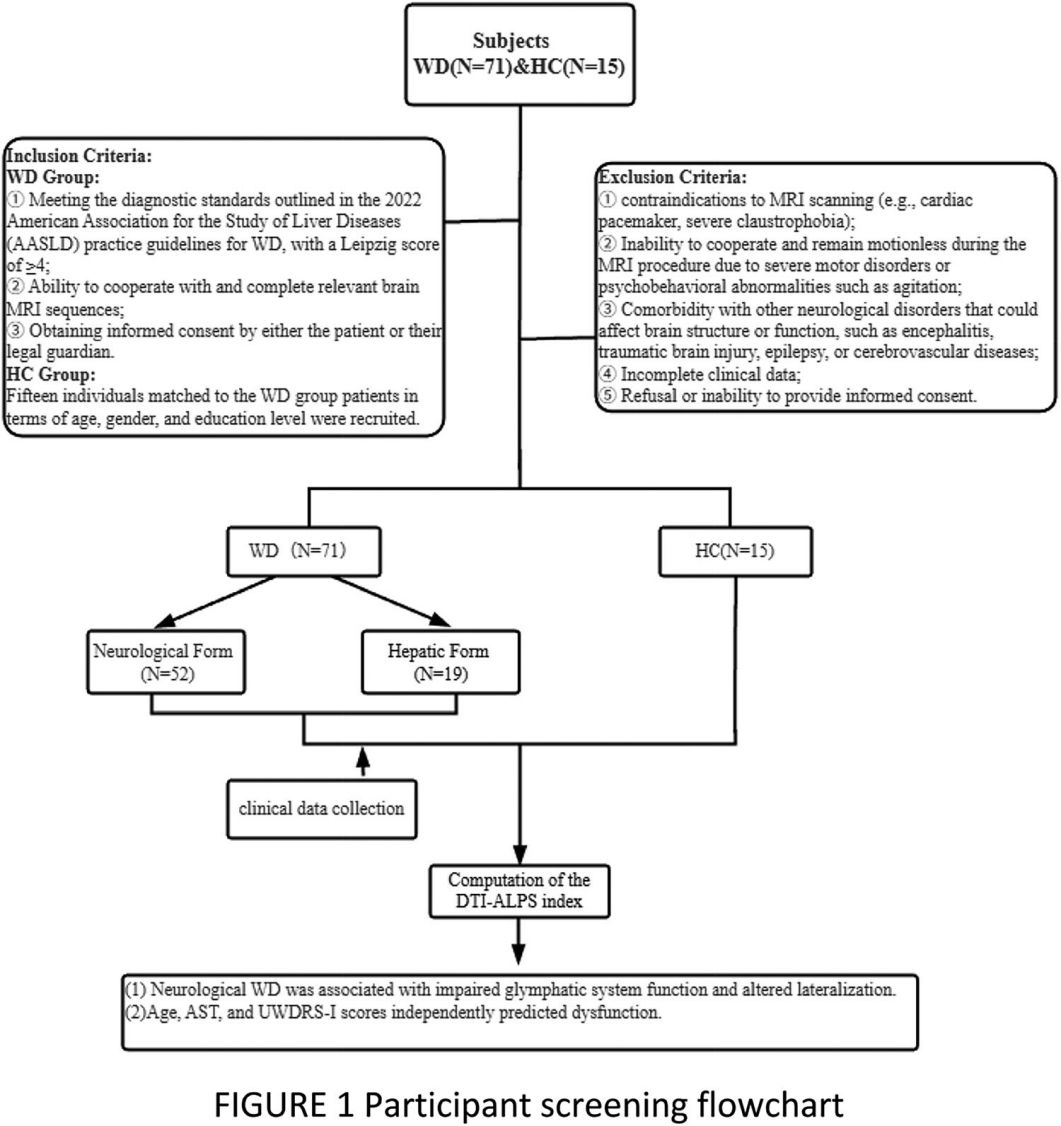
Participant screening flowchart.

### 2.2 Clinical Data Collection

Data on age, sex, and education level were collected from patients with WD and HCs. Additional data were collected from patients with WD, including abdominal color Doppler ultrasonography (Siemens, USA 802001), disease duration, and clinical phenotypes. Education level was categorized into four groups: primary school or lower, junior high school, technical secondary school or high school, and junior college or bachelor's degree. Liver ultrasonography findings were classified as cirrhotic or non‐cirrhotic. Clinical phenotypes of WD were classified according to the presence or absence of neurological symptoms at the time of enrollment. Patients with neurological symptoms attributable to WD were assigned to the neurological form group, whereas those without such symptoms were assigned to the hepatic form group.

### 2.3 Biochemical Indicator Assays

Following an 8‐hour overnight fast, venous blood samples were collected from all patients and analyzed the following morning. The following parameters were measured using a fully automated biochemical analyzer (Hitachi 7180): total bilirubin (TB), alanine aminotransferase (ALT), aspartate aminotransferase (AST), serum albumin (Alb), plasma ammonia (NH_3_), fasting blood glucose (FBG), and ceruloplasmin (Cp). Prothrombin time (PT) was determined using a fully automated coagulation analyzer (JY‐012). Before treatment, serum Cu and 24‐hour urinary Cu excretion (UC) were measured using a WFX‐120B atomic absorption spectrophotometer.

### 2.4 Scale Assessments

Neurological function was assessed using Part I (Neurological Examination) of the Chinese version of the unified WD rating scale (UWDRS). This version was developed through a rigorous translation and back‐translation process for cross‐cultural adaptation and has been demonstrated to have excellent reliability and validity (Han et al. 2013). Patients with WD and cirrhosis were evaluated using the Child‐Pugh classification criteria (Pugh et al. 1973), whereas non‐cirrhotic patients were assigned a score of zero. Within the Child‐Pugh criteria, hepatic encephalopathy was graded according to the West Haven criteria (Conn et al. 1977).

### 2.5 Brain Imaging Data Acquisition and Processing

Brain imaging data were acquired using a 1.5T MRI scanner (Siemens Medical Solutions, Erlangen, Germany). During scanning, participants lay supine on the examination table with their eyes closed, maintaining full‐body relaxation while refraining from falling asleep or engaging in deep thought. Each participant wore earplugs to reduce noise interference, and foam pads were used to secure both sides of the head to minimize motion‐related artifacts. Following proper placement of the head coil, the participant's head was positioned at the magnetic isocenter.

### 2.6 Scanning Sequences and Parameters

(1) 3D high‐resolution T1‐weighted structural imaging (3D‐T1WI): Acquired using a fast field echo pulse sequence. Parameters: repetition time (TR) = 2200.0 ms, echo time (TE) = 3.31 ms, field of view (FOV) = 250 mm × 250 mm, matrix size = 256 × 256, flip angle (FA) = 8°, slice thickness = 1.0 mm. A total of 128 contiguous axial slices were obtained; (2) T2‐weighted imaging (T2–WI): Acquired to exclude patients with visible encephalomalacia foci. Parameters: TR = 4670.0 ms, TE = 101 ms, FOV = 230 mm × 230 mm, matrix size = 256 × 256, FA = 150°, slice thickness = 5.0 mm. A total of 23 contiguous axial slices were obtained. (3) DTI: Performed using a single‐shot echo‐planar imaging sequence. Parameters: TR = 9600 ms, TE = 105 ms, FOV = 230 mm × 230 mm, matrix size = 128 × 128, FA = 90°, slice thickness = 2.0 mm. Data were acquired along 60 diffusion‐encoding directions with b‐values of 0 and 1000 s/mm^2^.

### 2.7 Image Data Processing and Analysis

DTI Data Preprocessing and ALPS Index Calculation: (1) format conversion: original DICOM files were converted to NIfTI format using MRIcroGL software. (2) image correction: NIfTI files were converted to SRC files using DSI Studio software. TOPUP/EDDY and motion correction were applied to improve image registration accuracy and fiber tracking fidelity; (3) data reconstruction: performed using the Q‐space diffeomorphic reconstruction (QSDR) method (Yeh and Tseng 2011), generating color‐coded fractional anisotropy (FA) and diffusion maps along the *x*‐, *y*‐, and *z*‐axes; (4) ROI delineation: projection and association fibers were identified on the color‐coded FA maps. Spherical regions of interest (ROIs) with a radius of 3 mm were placed bilaterally in the projection and association fiber regions at the level of the lateral ventricular body, yielding four ROIs; (5) data extraction and ALPS index calculation (Taoka et al. 2017): after ROI placement, Dxx, Dyy, and Dzz diffusion values were extracted from the bilateral projection and association fiber regions. These values were used to separately calculate the ALPS index for the left and right sides. The mean of the bilateral ALPS indices was then computed and used for subsequent statistical analyses.

Simultaneously, the V_WM/TIV was calculated using the 3D‐T1WI data. The processing pipeline was as follows: original DICOM files were converted to NIfTI format using the dcm2niix software. The NIfTI files were then imported into MATLAB, where spatial normalization, intensity correction, tissue segmentation, and volume calculations were performed using the SPM12 and CAT12 toolkits. The automatically generated report from the CAT12 toolbox was reviewed to exclude outlier data. Total white matter volume (V_WM) and total intracranial volume (TIV) were measured directly, and the V_WM/TIV ratio was calculated for subsequent statistical analyses.

### 2.8 Statistical Analysis

All data were analyzed using R software (version 4.5.1). The normality of continuous variables was assessed using the Shapiro‐Wilk test. Normally distributed continuous data are presented as mean ± standard deviation ($\bar{\chi }$ ± SD). Comparisons between two paired groups were performed using the paired t‐test, whereas comparisons across three groups were conducted using one‐way analysis of variance (ANOVA). Non‐normally distributed continuous data are presented as medians with interquartile ranges (*M* [Q1, Q3]). Comparisons between two independent groups were performed using the Mann–Whitney U test; comparisons between two paired groups were performed using the Wilcoxon signed‐rank test; and comparisons among three groups were performed using the Kruskal–Wallis H test. Categorical data are presented as frequencies (counts). Comparisons among three groups were performed using the *R* × *C* chi‐square test. When the conditions for the chi‐square test were not met, Fisher's exact test was employed. Correlation analyses were performed using Pearson or Spearman correlation analyses, as appropriate. Univariate and multivariate linear regression analyses were used to identify factors associated with the ALPS index. Figures were generated using R software (version 4.5.1). The significance level (α) was set at 0.05, and statistical significance was defined as a two‐tailed *p*‐value < 0.05.

## 3 Results

### 3.1 Comparison of Baseline Characteristics

A total of 71 patients with WD, including 19 with the hepatic form and 52 with the neurological form, as well as 15 HCs, were included in this study. No statistically significant differences were observed in age, sex, or education level among the three groups (all *p* > 0.05). Furthermore, there was no significant difference in disease duration between the hepatic and neurological forms (*p* > 0.05). These findings indicated that the groups were comparable at baseline (Table 1).

**TABLE 1:** Basic information.

Characteristics	Neurological form group	Hepatic form group	HCs	Statistical values	*p* value
	(*N* = 52)	(*N* = 19)	(*N* = 15)		
Age(yr)	29.0(21.5–34.0)	22.0(20.0–33.0)	28.0(25.0–31.0)	*H* = 3.510	0.173
Disease duration(mos.)	162.0(60.0–228.0)	132.0(84.0–180.0)	—	*U* = 459.000	0.649
Gender	—	—	—	*χ* ^2^ = 2.798	0.252
Male	31	8	6	—	—
Female	21	11	9	—	—
Education level	—	—	—	*χ* ^2^ = 10.165	0.087
Primary school or lower	3	1	2	—	—
Junior high school	9	3	1	—	—
Technical secondary school or high school	21	2	3	—	—
Junior college or bachelor's degree	19	13	9	—	—

### 3.2 Comparison of ALPS Index

#### 3.2.1 Intergroup Comparisons

The ALPS index was significantly lower in the neurological form group (1.44 ± 0.14) compared with both the hepatic form group (1.53 ± 0.16) and the HC group (1.62 ± 0.12) (*p* < 0.05 for both). Although the ALPS index in the hepatic form group was lower than that in the HC group, this difference was not statistically significant (*p* > 0.05) (Figure 2).

**FIGURE 2:**
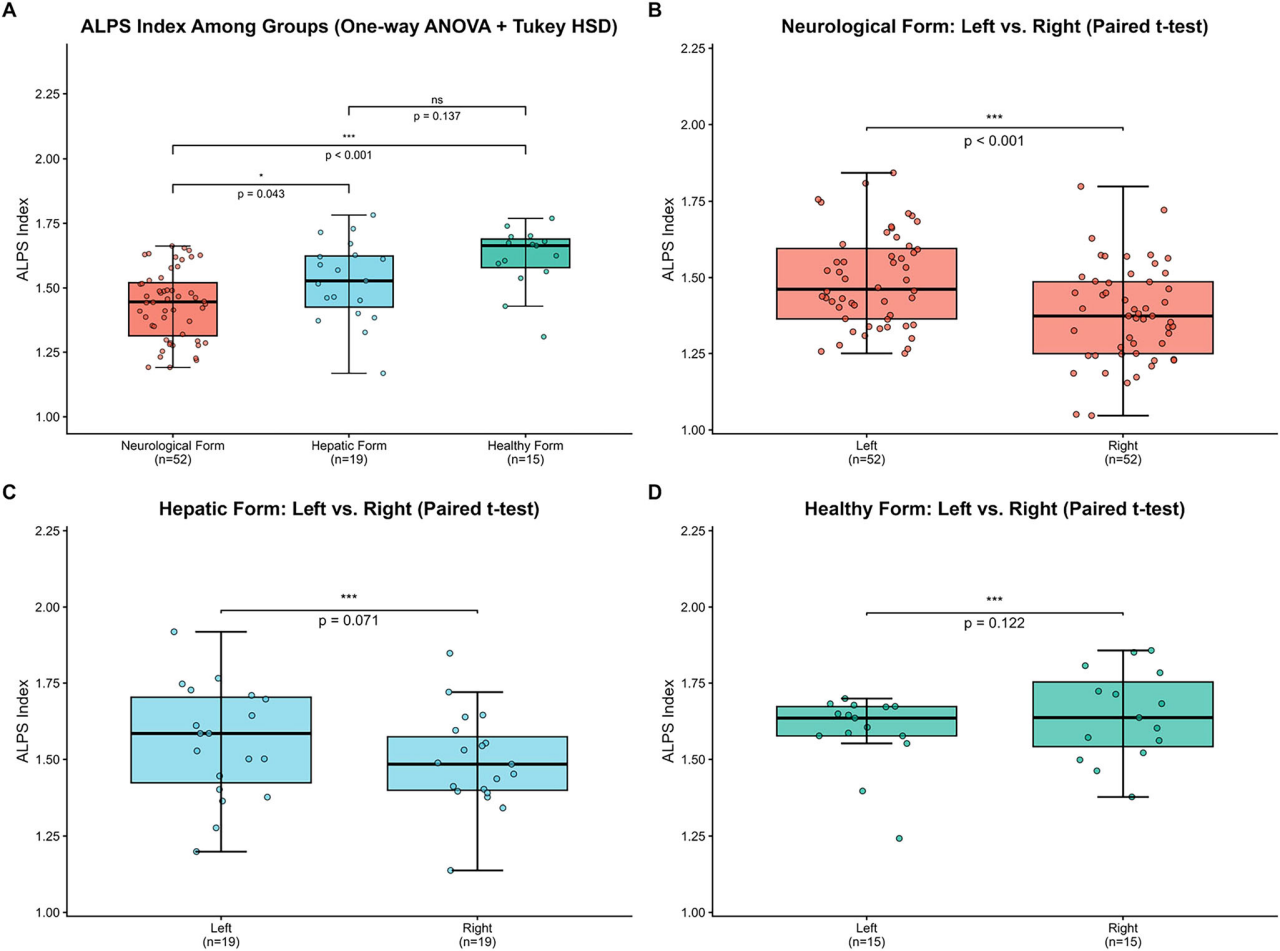
Comparison of ALPS index across different subgroups.

#### 3.2.2 Intragroup Comparisons

Within the neurological form group, the left‐sided ALPS index (1.49 ± 0.15) was significantly higher than the right‐sided index (1.38 ± 0.16) (*p* < 0.001). In contrast, no significant left‐right differences were observed in either the hepatic form or HC group (*p* > 0.05) (Figure 2). To further compare the degree of interhemispheric asymmetry among the three groups, we calculated the left‐right difference (ght difference (ht diffe− right ALPS index) for each participant. Relative to the right side, the interhemispheric difference was 8.0% in the neurological form group (left: 1.49 ± 0.15, right: 1.38 ± 0.16), 4.7% in the hepatic form group (left: 1.56 ± 0.04, right: 1.49 ± 0.04), and 3.1% in the HC group (left: 1.64 % in the neurological form group (left: 1.49 ± 0.15, right: 1.38 ± 0.16), ce in ΔALPS among the three groups (*p* = 0.001). Post‐hoc LSD tests further showed that the rological form group (left: 1.49 ± 0.15, right: 1.38 ± 0.16), ce in ΔALPS amoHC group (*p* < 0.001), and the ed that the rological form group (left: 1.4er than that in the HC group (*p* = 0.026), whereas the difference between the neurological WD and hepatic WD groups was not statistically significant (*p* = 0.210).

### 3.3 Correlation Analysis

Correlation analysis of potential influencing factors in the neurological form group revealed significant positive correlations between the ALPS index and Alb level (*r* = 0.278, *p* = 0.046), FBG level (*r* = 0.287, *p* = 0.039), and V_WM/TIV ratio (*r* = 0.342, *p* = 0.013). Conversely, significant negative correlations were found with age (*r* = ‐0.277, *p* = 0.047), ALT level (*r* = ‐0.309, *p* = 0.026), AST level (*r* = ‐0.408, *p* = 0.003), and UWDRS‐I score (*r* = ‐0.484, *p* < 0.001). No significant correlations were observed with sex, education level, Cu level, Cp level, UC value, TB level, disease duration, Child‐Pugh grade, or ultrasound classification of the Liver (all *p* > 0.05) (Figure 3).

**FIGURE 3:**
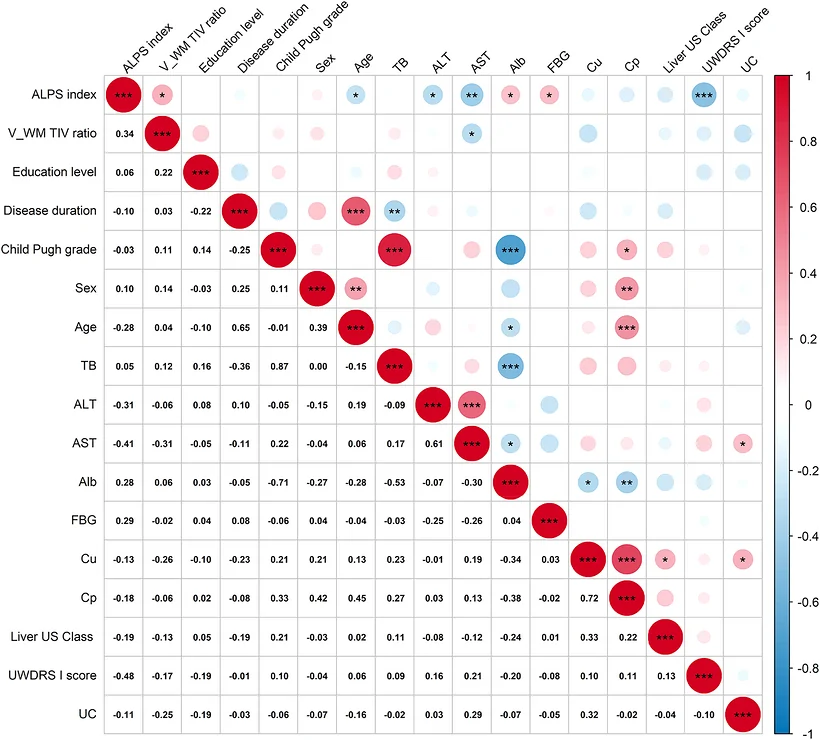
Correlation Heatmap.

### 3.4 Simple Linear Regression Analysis

Simple Linear Regression Analysis was performed using the ALPS index of patients with the neurological form as the dependent variable. Independent variables included sex, education level, age, disease duration, TB, Alb, ALT, AST, FBG, Cu, Cp, UC, UWDRS‐I score, V_WM/TIV ratio, Child‐Pugh grade, and liver ultrasonography classification. Education level and Child‐Pugh grade were treated as dummy variables. Each independent variable was entered into a separate univariate regression model. Results indicated that Alb level, FBG level, and V_WM/TIV ratio were significant positive predictors of the ALPS index (*p* < 0.05), whereas age, ALT level, AST level, and UWDRS‐I score were significant negative predictors (*p* < 0.05).

### 3.5 Multiple Linear Regression Analysis

Multiple linear regression analysis was performed using the ALPS index of patients in the neurological form group as the dependent variable. Independent variables showing significant associations in univariate analysis (age, Alb level, FBG level, V_WM/TIV ratio, ALT level, AST level, and UWDRS‐I score) were included in the multivariate model. The results demonstrated that age, AST level, and UWDRS‐I score were independent negative predictors of the ALPS index in patients with neurological WD (Figure 4). The resulting regression equation was: ALPS index = 1.67 − 0.003 × UWDRS‐I score − 0.002 × AST level − 0.004 × age (years). The model yielded an adjusted *R^2^* of 0.350 and a Durbin–Watson statistic of 1.827, indicating good effectiveness and robustness.

**FIGURE 4:**
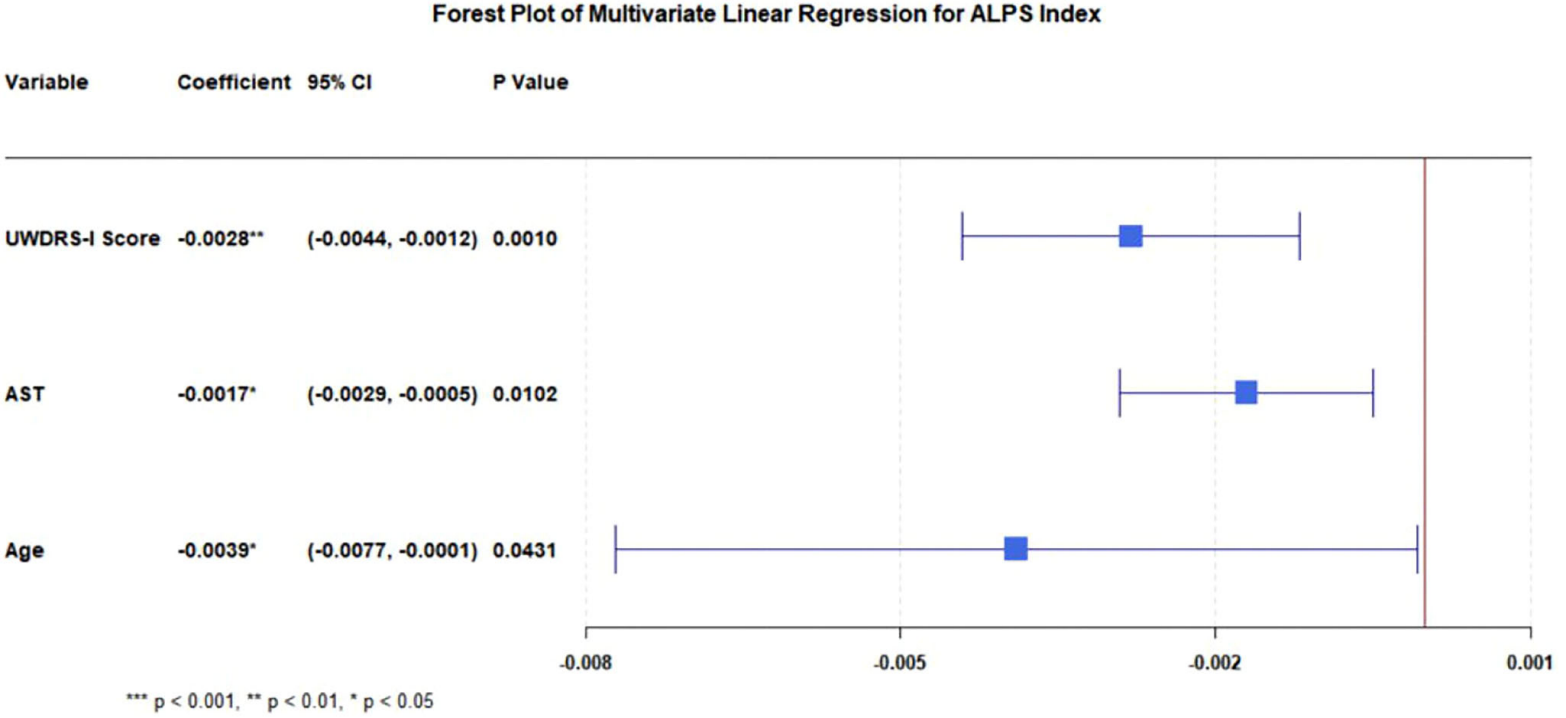
Forest plot of multiple linear regression analysis for ALPS index.

## 4 Discussion

This study was the first to employ DTI–ALPS to evaluate glymphatic system function in patients with WD and to preliminarily explore the risk factors for glymphatic dysfunction in this population. The principal findings were as follows: (Sandahl et al. 2020) Compared with HCs, patients with the neurological form of WD exhibited significant glymphatic system dysfunction, which was more pronounced on the right side; these alterations were absent in patients with the hepatic form. (Hu et al. 2011) In the neurological WD cohort, the ALPS index showed significant positive correlations with Alb level, FBG level, and the V_WM/TIV ratio, whereas it correlated negatively with age, ALT level, AST level, and UWDRS‐I score. (Hu et al. 2012) Multiple analysis further identified older age, elevated AST levels, and more severe neurological symptoms as independent risk factors for glymphatic dysfunction in neurological WD.

The most critical finding of this study is that it provides the first evidence implicating glymphatic system dysfunction in the pathogenesis of the neurological form of WD. Neurological damage in WD primarily results from systemic Cu toxicity and involves multiple pathological mechanisms. Animal studies of Cu toxicity have demonstrated that increased brain Cu levels lead to its accumulation within astrocytes, suggesting a protective role for these cells in sequestering excess Cu to shield neurons (Haywood et al. 2008). However, as cerebral Cu accumulation progresses, astrocytes undergo proliferation, hypertrophy, and pathological morphological changes, giving rise to Type I and Type II Alzheimer's cells and Opalski's cells (Mossakowski et al. 1970; Bertrand et al. 2001; Gromadzka et al. 2024). Postmortem examinations of the brain of patients with neurological WD have revealed neuronal loss, glial cell proliferation, and enlarged VRS (Meenakshi‐Sundaram et al. 2008). In the WD brain, pathological alterations, including astrocytic proliferation, dysplasia, and enlarged VRS, directly compromise the anatomical architecture of the glymphatic system. This structural disruption, in turn, impairs clearance. The resulting glymphatic dysfunction is expected to hinder Cu elimination from the brain, thereby establishing a self‐reinforcing vicious cycle that exacerbates disease progression. This cycle has been proposed as the key pathological basis for glymphatic dysfunction in neurological WD. In contrast, hepatic WD is characterized predominantly by Cu deposition and resultant liver damage, whereas structures such as cerebral astrocytes remain largely unaffected. This fundamental distinction explains the preserved glymphatic function observed in these patients.

Our study revealed that glymphatic dysfunction in the left hemisphere was relatively milder in neurological WD patients, as evidenced by a significantly higher left ALPS index compared to the right, with an interhemispheric difference of 8.0%, indicating a pronounced lateralization pattern. This may be related to the functional and structural lateralization of the human brain (Ocklenburg et al. 2024). Under normal conditions, the left hemisphere is closely associated with language, executive function, and higher‐order cognitive processes, and this lateralization may necessitate greater metabolic activity and glymphatic clearance capacity in the left hemisphere. Under pathological conditions, the left hemispheric glymphatic system, possessing greater functional reserve and compensatory capacity, may be more resistant to pathological insults. This could be one of the key reasons why neurological WD patients are more susceptible to right‐sided glymphatic dysfunction while left‐sided function remains relatively preserved. Similar lateralized changes have also been reported in Parkinson's disease (van et al. 2012). Furthermore, it is noteworthy that although the ALPS index in hepatic WD patients did not differ significantly from that of HCs, the left‐right difference was significantly greater than that in controls, suggesting that alterations in interhemispheric glymphatic asymmetry may represent an imaging change that precedes a detectable decline in the ALPS index.

In the correlation analysis, positive associations between the ALPS index and both Alb and FBG levels in patients with neurological WD suggested a close relationship between systemic nutritional status and glymphatic system function. Serum Alb, synthesized by the liver, is crucial for maintaining overall nutritional status and plasma colloid osmotic pressure. A decrease in serum Alb levels in patients with neurological WD reduces plasma colloid osmotic pressure, which may alter osmotic pressure dynamics within the glymphatic fluid and consequently impair glymphatic system function. Conversely, higher serum Alb levels are likely to exert a protective effect, resulting in a lower impact on glymphatic function. This could explain the positive correlation between the ALPS index and serum Alb levels, although this hypothesis requires further clinical validation. Plasma glucose (FBG) serves as the primary energy source for brain astrocytes. Studies have shown that under conditions such as ischemia or impaired energy supply, astrocytes undergo reactive proliferation and lose normal physiological function (Sofroniew and Vinters 2010). Adequate FBG levels are essential for maintaining astrocytic function and structural integrity, which likely underlies the observed positive correlation between the ALPS index and FBG levels. The structural integrity of cerebral white matter provides the anatomical foundation for glymphatic pathway patency. The V_WM/TIV ratio primarily reflects the degree of white matter preservation. A higher V_WM/TIV ratio indicates more intact white matter architecture, which facilitates unobstructed glymphatic pathways and enhances clearance function. This mechanistic relationship explains the observed positive correlation between the ALPS index and V_WM/TIV ratio.

In contrast, negative correlations were observed between the ALPS index and ALT and AST levels in patients with neurological WD. ALT and AST are key biochemical markers of hepatocellular injury, and their elevation reflects the aggravation of liver damage. Previous studies have shown that liver diseases of various etiologies are associated with inflammatory responses, and the degree of inflammation intensifies with increasing severity of liver injury (Costa et al. 2021; De Carvalho Ribeiro and Szabo 2022; Taru et al. 2024). Meanwhile, experimental evidence suggests that inflammation can impair glymphatic function through mechanisms such as obstructing cerebrospinal fluid flow in the perivascular spaces and downregulating AQP4 expression in astrocytes (Cai et al. 2024). Based on the above evidence, we hypothesize that the negative correlation between ALT/AST levels and the ALPS index in neurological WD patients may be partially mediated through inflammatory pathways via the “liver‐brain axis” (D‘Mello and Swain 2011; Mikkelsen et al. 2025). However, given that inflammatory cytokines or AQP4 expression levels were not measured in the present study, this hypothesis warrants further investigation. Finally, we observed a strong negative correlation between the ALPS index and the UWDRS‐I score in patients with neurological WD. The UWDRS, developed by Leinweber et al. to assess the clinical severity of WD, demonstrates good reliability and validity (Leinweber et al. 2008). The Chinese version of the UWDRS has been validated in the Chinese population, confirming sound psychometric properties for clinical assessment, with Part I specifically designed to evaluate neurological function (Han et al. 2013). Higher UWDRS‐I scores directly reflect more severe brain injury in patients with WD, which is concomitantly associated with greater glymphatic system dysfunction.

Preliminary correlation analysis revealed multiple factors influencing glymphatic system function. To identify more robustly associated factors among these variables, we performed a multiple linear regression analysis. The results demonstrated that only age, AST level, and the UWDRS‐I score were independent risk factors for glymphatic dysfunction in patients with the neurological form of WD. Among these, advanced age is a well‐established factor contributing to the physiological decline of glymphatic function. The underlying mechanisms are closely linked to age‐related reductions in arterial pulsatility and downregulation of astrocytic AQP4 expression (Voumvourakis et al. 2023; Xiong et al. 2024). The present study revealed that age was an independent negative associated factor of the ALPS index in neurological WD patients, and this association remained significant after controlling for disease severity (UWDRS‐I) and liver injury (AST). Considering that the ALPS index in hepatic WD patients did not differ significantly from that of HCs, whereas a marked reduction was observed in neurological WD patients, the detrimental effect of age on glymphatic function does not appear to be a universal phenomenon across all WD patients, but rather emerges specifically in those with neurological involvement, suggesting that neurological damage may serve as an important synergistic factor accelerating age‐related glymphatic decline. AST is primarily enriched in hepatocyte mitochondria, and its elevation often indicates severe hepatocellular injury. Its identification as a factor independently associated with glymphatic dysfunction suggests that liver damage in patients with neurological WD may negatively impact the glymphatic system, potentially via a “liver‐brain axis” pathway. Furthermore, the UWDRS‐I score as an independent risk factor strongly supports a direct and close pathophysiological link between the severity of neurological symptoms and the degree of glymphatic system impairment. Notably, although univariate analysis revealed a significant association between V_WM/TIV ratio and the ALPS index, this effect was no longer significant after controlling for age, AST, and UWDRS‐I score. This suggests that the association between white matter atrophy and glymphatic dysfunction may be largely mediated by disease severity, and that these two measures may represent parallel pathological processes rather than a causal relationship. As a functional imaging metric reflecting brain functional status, the ALPS index complements clinical indicators and may contribute to a more comprehensive assessment of brain involvement in WD patients.

## 5 Conclusion

In summary, this study elucidated the characteristics of glymphatic system function in patients with WD, revealing significant impairment in those with the neurological form. The ALPS index showed potential as a practical biomarker for phenotype differentiation, longitudinal monitoring in hepatic WD, and treatment response assessment. Furthermore, we identified older age, elevated AST levels, and higher UWDRS‐I scores as factors independently associated with glymphatic system dysfunction in neurological WD, providing clinicians with a concise and robust risk assessment framework to identify high‐risk individuals. However, this study has several limitations. First, its cross‐sectional design precludes causal inferences regarding the relationship between glymphatic system dysfunction and disease progression. Second, as a single‐center study with relatively small sample sizes in certain subgroups, the generalizability of these findings may be limited and requires validation through large‐scale multicenter investigations. Third, treatment‐related variables were not incorporated into the analysis, and their potential impact warrants further investigation. Lastly, although we utilized the non‐invasive and convenient ALPS index to indirectly assess glymphatic function, it has not been directly validated against the tracer‐based gold standard. Future studies should therefore involve direct comparative analyses of these two methodologies in a TX mouse model.

## Author Contributions

**Liang‐jie Zhang**: software, visualization. Tong Wu: software, Writing – original draft. Bin Song: investigation, resources. **Ben‐chun Xue**: project administration. Long Zhang: investigation, resources. Quan Sun: supervision. **Yu‐long Zhu**: investigation, resources. Lei Hua: investigation, resources. **Qin‐yuan Liu**: investigation, resources. **Liang‐liang Zhang**: investigation, resources. Bo Li: investigation, resources, data curation. **Yong‐sheng Han**: funding acquisition, writing – review and editing, project administration, supervision. Ling Zhu: writing – review and editing, writing – original draft, software. Yin Xu: supervision, validation. **Yong‐zhu Han**: project administration, supervision, writing – review and editing.

## Funding

This project is funded by the Modernization of Traditional Chinese Medicine of IHM (2023CXMMTCM002), Anhui Provincial Clinical Translational Special Project: Research on Integrated Evaluation Model and Key Technology of “Disease Evidence‐Procedure‐Effect” of Hepatolenticular degeneration by Combination of Traditional Chinese and Western Medicine and Prevention (202204295107020047).

## Ethics Statement

The study protocol was established, according to the ethical guidelines of the Helsinki Declaration and was approved by the ethics committee of the hospital (2023‐SYSFYSY‐32).

## Conflicts of Interest

The authors declare no conflicts of interests.

## Data Availability

The data that support the findings of this study are available on request from the corresponding author. The data are not publicly available due to privacy or ethical restrictions.
